# Surgical cryoprobe ablation for premature ventricular contraction of left ventricular summit refractory to catheter ablation

**DOI:** 10.1002/joa3.70113

**Published:** 2025-06-16

**Authors:** Yasuyuki Egami, Masamichi Yano, Naotaka Okamoto, Yasuharu Matsunaga‐Lee, Masami Nishino, Haruhiko Kondoh

**Affiliations:** ^1^ Division of Cardiology Osaka Rosai Hospital Sakai Osaka Japan; ^2^ Division of Cardiovascular Surgery Osaka Rosai Hospital Sakai Osaka Japan

**Keywords:** left ventricular summit, premature ventricular contraction, refractory to catheter ablation, surgical cryoablation

## Abstract

The successful elimination of left ventricular (LV) summit premature ventricular contractions (PVCs) using surgical endocardial cryoablation. Guided by preoperative activation mapping, cryoablation was performed during aortic valve replacement without epicardial access. This approach effectively resolved PVCs refractory to catheter ablation.

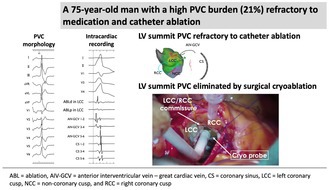

The left ventricular (LV) summit is a challenging site for catheter ablation (CA) of premature ventricular contractions (PVCs) due to its proximity to major coronary vessels and thick epicardial fat, even when approached from various sites, including the coronary cusp, great cardiac vein‐anterior interventricular vein junction, basal LV endocardium, and right ventricular outflow tract.^1^ Despite using the epicardial approach, CA outcomes for PVCs originating from the LV summit have remained unsatisfactory.^2^

The patient was a 75‐year‐old man planned to undergo aortic valve replacement (AVR) for severe aortic regurgitation. CA was performed prior to surgery because of high PVC burden (21% on 24‐h Holter ECG), which was refractory to antiarrhythmic drug and associated with symptoms such as exertional fatigue. The morphology of target PVC is shown in Figure 1A. Intracardiac recordings identified the earliest activation site of the PVCs at the distal electrode of the anterior interventricular vein—great cardiac vein catheter (Figures 1B,C and 2), preceded any potential recorded in the left coronary cusp. Activation map with CARTO3 system (Biosense Webster, Diamond Bar, CA, USA) displayed the earliest activation site as being around the LCC (Figure 3A–B). Radiofrequency applications were delivered using THERMOCOOL SMARTTOUCH® SF (Biosense Webster) catheter at a power of 30–35 W for 60 s from the left coronary cusp, right coronary cusp, and beneath the aortic valve as part of a stepwise anatomical approach (Figure 3C). To minimize stress on the aortic valve during catheter insertion into the LV, intracardiac echocardiography was used for precise guidance. Ablation was performed after confirming that the tip of the ablation catheter was positioned away from the aortic valve leaflets to avoid exacerbation of aortic regurgitation due to catheter‐induced trauma. Although these radiofrequency applications transiently suppressed the PVCs, they ultimately failed to eliminate them.

**FIGURE 1:**
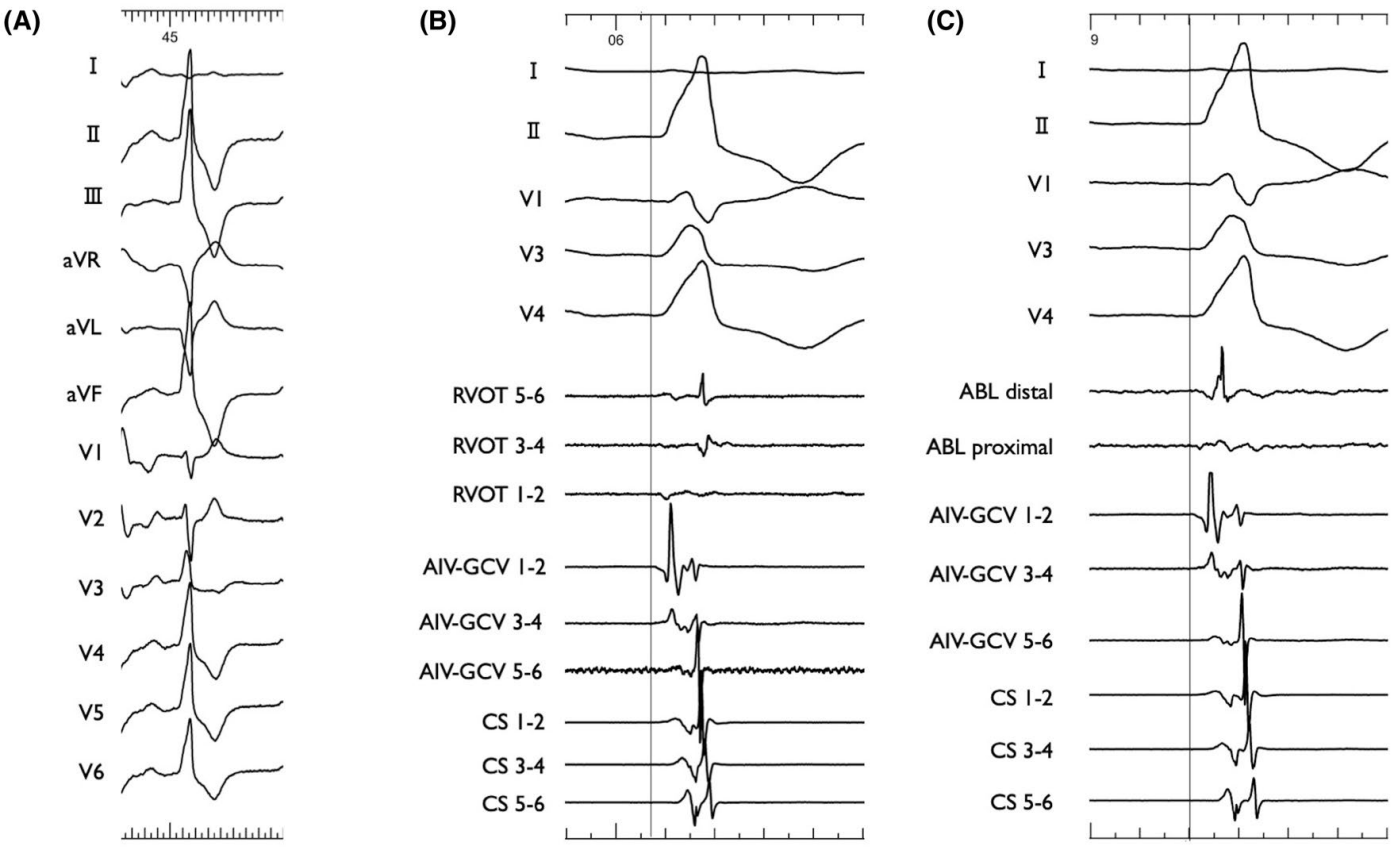
(A) Twelve‐lead ECG of the clinical PVC. (B) Intracardiac electrogram during PVC from multiple sites including the AIV‐GCV and RVOT. (C) Intracardiac electrogram showing the ablation catheter positioned at the LCC. The vertical line indicates the site of the earliest activation. ABL, ablation; AIV‐GCV, anterior interventricular vein‐great cardiac vein; CS, coronary sinus; LCC, left coronary cusp; PVC, premature ventricular contraction; RVOT, right ventricular outflow tract.

**FIGURE 2:**
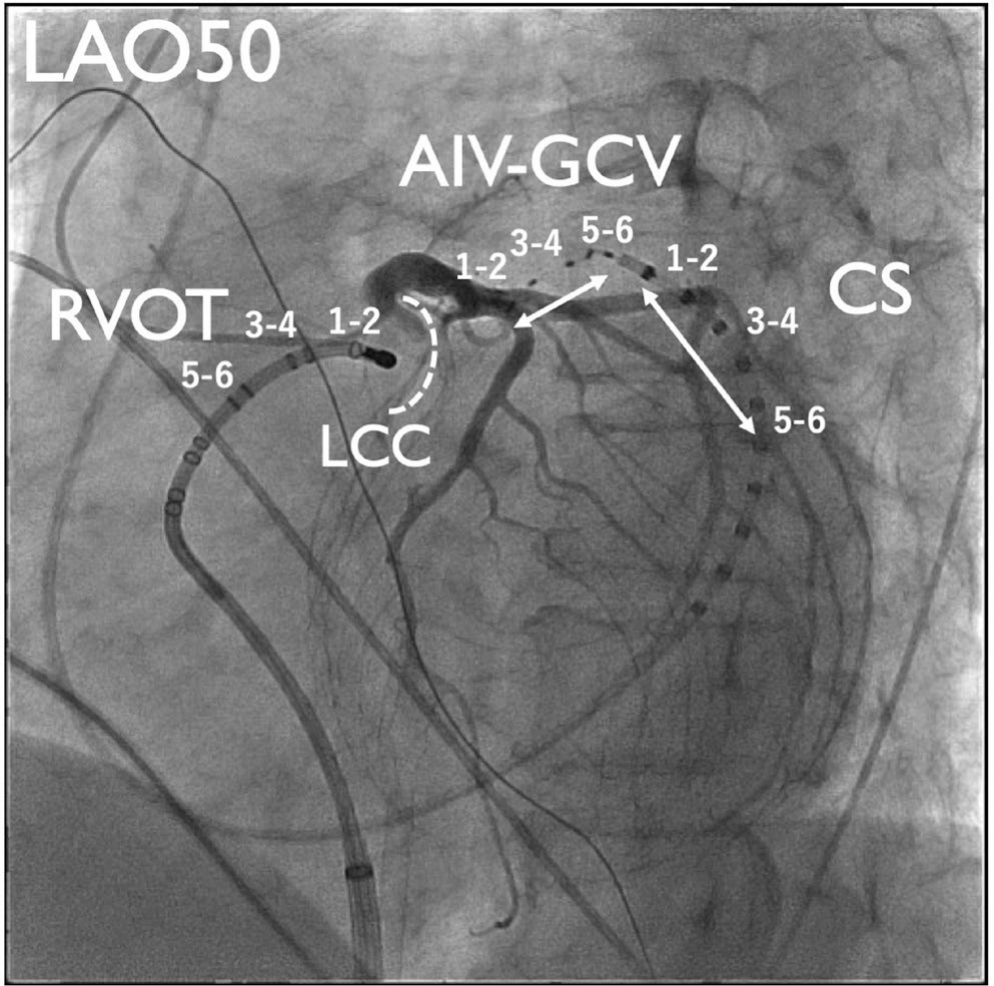
Fluoroscopic images in the LAO projections showing the catheter positions. Dotted curved line outlines the contrasted LCC. LAO, left anterior oblique. The other abbreviations are shown in Figure 1.

**FIGURE 3:**
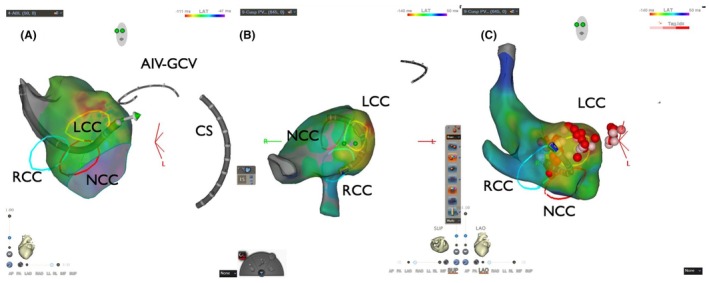
Three‐dimensional electro‐anatomical activation map of PVC. (A) LAO view, (B) superior view, and (C) ablation sites (colored circle tags) Yellow, blue, and red circles represent the LCC, right coronary cusp (RCC), and noncoronary cusp (NCC), respectively. The other abbreviations are shown in Figure 1.

Six months later, using the previous activation map image of the PVC as a reference (Figure 3), surgical endocardial cryoablation (CCS‐200, Cooper Surgical, Shelton, CT, USA) was performed just prior to the AVR. During cardiac arrest, two cryoablation applications of 120 s each were delivered at a target temperature of −60°C beneath the left and right coronary cusps (Figure 4). Although epicardial ablation is frequently required to achieve transmural lesions in the LV summit,^3^ in our case, ablations from not only the left coronary cusp but also the LV endocardium transiently eliminated the target PVCs, suggesting that the arrhythmogenic focus could potentially be eliminated from the endocardial surface. Given the proximity to the left main trunk and the size of the cryoprobe (25 mm × 5 mm), epicardial surgical cryoablation was avoided to minimize the risk of coronary injury. No PVC recurrence was observed on 24‐h Holter ECG at 12 months after the AVR procedure without any antiarrhythmic drugs.

**FIGURE 4:**
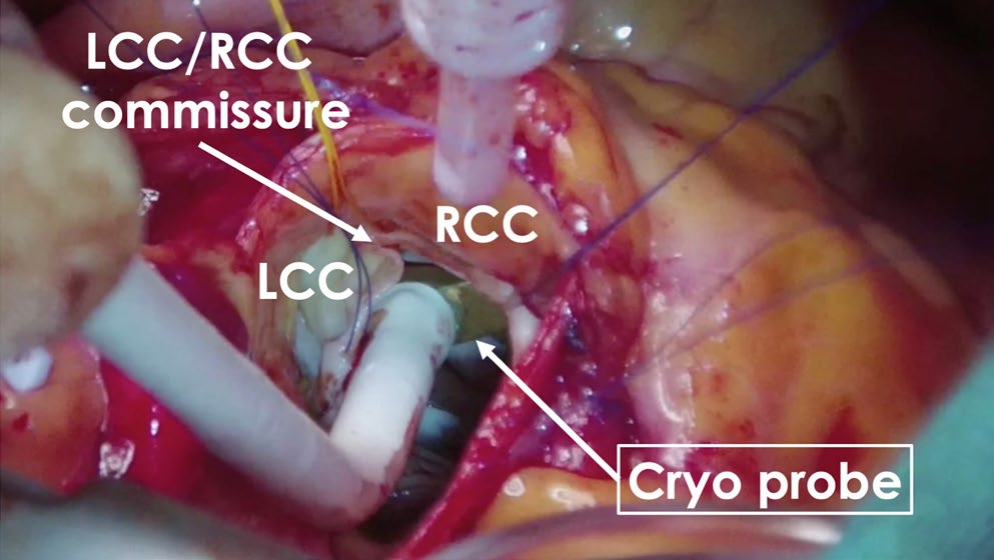
Surgical endocardial cryoablation site. The cryo probe was positioned beneath the LCC and RCC. The width and depth of the cryo probe are 25 mm and 5 mm, respectively. Abbreviations are the same as Figures 1 and 3.

The LV summit is the most superior region of the LV epicardium, located between the left anterior descending artery (proximal to the first septal perforator), the left circumflex artery, and the great cardiac vein/anterior interventricular vein. Clinically significant for idiopathic ventricular arrhythmias, it is often considered an inaccessible region for CA due to its proximity to major coronary arteries and the great cardiac vein, making arrhythmia treatment challenging.^1^ Cryoablation for ventricular arrhythmias near the proximal left anterior descending artery, refractory to percutaneous endocardial and epicardial approaches, has been successfully performed using a minimally invasive surgical approach with robotic‐assisted mapping and mini‐thoracotomy.^4^ Anter et al. reported that ventricular arrhythmia refractory to percutaneous CA in patients with nonischemic substrate can be successfully treated when guided by prior detailed electroanatomic and electrophysiological mapping.^5^ In cases where PVCs require surgical intervention, an epicardial approach is typically considered. However, this case highlights that surgical cryoablation from the endocardium, guided by a prior electroanatomical map image of target PVCs, can be effective even when initial endocardial CA for PVCs of the LV summit is unsuccessful. In cases of postoperative recurrence, alternative strategies such as an antegrade approach via transseptal puncture site or epicardial surgical approach may be considered.

## FUNDING INFORMATION

None.

## CONFLICT OF INTEREST STATEMENT

None.

## ETHICS STATEMENT

This paper complies with the ethics and integrity policies of *Journal of Arrhythmia*.

## PATIENT CONSENT STATEMENT

Obtained from the patient.

## CLINICAL TRIAL REGISTRATION

N/A.

## PERMISSION TO REPRODUCE MATERIAL FROM OTHER SOURCES

N/A.

## Data Availability

The data analyzed in the present case is available from the corresponding author upon reasonable request.
